# Liver microenvironment-driven immunosuppressive niche in colorectal cancer liver metastasis and its therapeutic remodeling

**DOI:** 10.3389/fimmu.2026.1906870

**Published:** 2026-08-18

**Authors:** Zijing Liu, Jia Liu, Shengshan Xu, Yumin Zhong, Ke-Jie He, Zhuming Lu, Youbin Zheng, Yi Zhang, Qian Guo, Hongyu Chu

**Affiliations:** 1 Department of Gastrointestinal and Colorectal Surgery, China-Japan Union Hospital of Jilin University, Changchun, China; 2 College of Landscape Architecture & Arts, Northwest A&F University, Xianyang, China; 3 Department of Thoracic Surgery, Jiangmen Central Hospital, Jiangmen, Guangdong, China; 4 China-Japan Union Hospital of Jilin University, Changchun, China; 5 The Quzhou Affiliated Hospital of Wenzhou Medical University, Quzhou People’s Hospital, Quzhou, Zhejiang, China; 6 Department of Radiology, Affiliated Jiangmen Traditional Chinese Medicine Hospital of Jinan University, Jiangmen, Guangdong, China; 7 Department of Rhinology, The First Affiliated Hospital of Zhengzhou University, Zhengzhou, China

**Keywords:** colorectal cancer liver metastasis, hepatic sink effect, immune checkpoint blockade, liver microenvironment, organ-specific resistance

## Abstract

Colorectal cancer liver metastasis is the most frequent and clinically important pattern of distant spread in colorectal cancer and remains a major determinant of poor prognosis and treatment failure. In the era of immunotherapy, liver metastasis has emerged as an organ-specific barrier to effective antitumor immune responses. Clinical and translational evidence suggests that this pattern of resistance is observed across both MSI-H/dMMR and MSS/pMMR disease contexts, highlighting the liver metastatic microenvironment as a consistent modifier of immunotherapy response. However, the biological mechanisms by which the liver metastatic environment shapes immunotherapy resistance have not been fully integrated, and the extent to which local hepatic immune suppression influences systemic antitumor immunity remains incompletely defined. This review summarizes the liver-specific biological basis of immunotherapy resistance in colorectal cancer liver metastasis, focusing on three interconnected mechanistic layers, including the pre-existing tolerogenic immune niche of the liver, the local immunosuppressive microenvironment established within liver metastases, and the systemic immune suppression associated with the hepatic sink effect. We further discuss therapeutic strategies aimed at enhancing tumor immunogenicity, reprogramming suppressive myeloid and dendritic cells, restoring the function of CD8^+^ T cells and innate-like effector lymphocytes, remodeling stromal and vascular barriers, and counteracting liver metastasis-driven systemic immune dysfunction. Future clinical and translational studies should prioritize liver-lesion-specific efficacy, paired primary and metastatic tissue analyses, assessment of liver metastatic burden and activity, and integrated immune biomarkers. A shift from a primary-tumor-centered view toward a liver-microenvironment-oriented framework may provide new opportunities to improve immunotherapy outcomes in patients with colorectal cancer liver metastasis.

## 1 Introduction

Colorectal cancer (CRC) is the third most commonly diagnosed malignancy and the second leading cause of cancer-related death worldwide, and metastatic CRC (mCRC) carries a markedly worse prognosis (1, 2). The liver is the most frequent metastatic site, with colorectal cancer liver metastasis (CRLM) occurring in nearly half of patients (3). Resectable CRLM is treated by surgical resection but recurs frequently (4), and unresectable disease relies on systemic therapy (5, 6). Immunotherapy has provided new opportunities in CRC (6). However, its efficacy in CRLM remains heterogeneous and limited, suggesting that liver metastases involve distinct mechanisms of resistance.

The response of CRLM to immunotherapy is first constrained by the molecular and immune subtype of the underlying CRC (7). Immune checkpoint inhibitors (ICIs) have become an important treatment modality for mismatch repair-deficient (dMMR) or microsatellite instability-high (MSI-H) mCRC (5, 8). These tumors carry a high mutational burden, abundant neoantigens, and an immune-hot phenotype (8, 9). However, approximately 95% of mCRC cases are mismatch repair-proficient (pMMR) or microsatellite stable (MSS) (10, 11). These tumors have lower mutational burden, fewer neoantigens, sparse effector T-cell infiltration, and an overall immune-cold phenotype, resulting in limited responses to ICIs (12). The benefit of immunotherapy in CRLM is therefore first restricted by the predominance of MSS disease.

Beyond the constraint imposed by tumor immune subtype, liver metastasis is consistently associated with reduced ICI efficacy in both MSI-H/dMMR and MSS/pMMR disease (13). In patients with MSI-H disease, progression-free survival (PFS) after first-line immunotherapy is markedly shortened when liver metastases are present (14). The same pattern is more pronounced in MSS disease, where the objective response rate (ORR) in patients with active liver metastases may approach zero (15). This consistency across molecular subtypes points to an organ-specific influence of the hepatic microenvironment and supports targeting the liver metastatic niche itself. Preclinical models provide more direct evidence. In mice simultaneously bearing subcutaneous and hepatic tumors, hepatic tumors render the distant subcutaneous tumors unresponsive to immunotherapy, while lung tumors of comparable burden leave the response intact (16). This organ specificity originates from the intrinsic immune tolerance of the liver, which is continuously exposed to gut-derived antigens and forms a tolerogenic niche before tumor colonization (17, 18). When CRC cells colonize this environment, local immune suppression develops within the metastatic lesion across multiple immune cell populations (18). Moreover, the immunosuppressive impact of liver metastases is not confined to local lesions; it can weaken systemic antitumor immunity against extrahepatic lesions, including through the elimination of activated effector T cells, thereby forming a hepatic sink effect (13).

**Figure 1:**
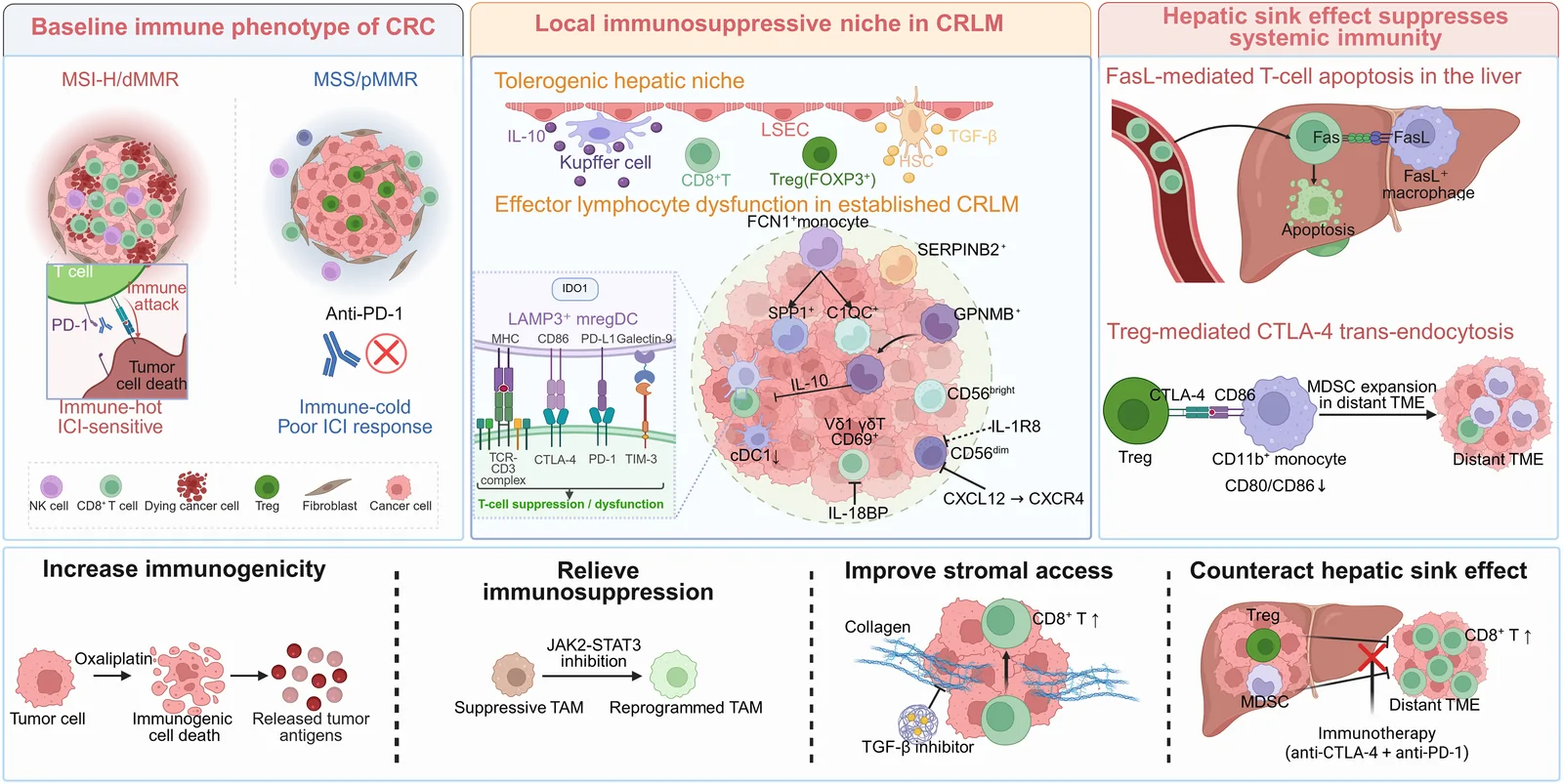
Liver microenvironment-driven immune resistance in colorectal cancer liver metastasis and its therapeutic remodeling. The image sets out the three mechanistic layers of organ-specific resistance, resolves the dendritic cell, effector lymphocyte, and tumor-associated macrophage compartments of the liver metastatic niche, and maps the therapeutic remodeling strategies onto the layers they target. Created with BioRender.com.

On this basis, this review summarizes the mechanistic foundation by which the liver microenvironment drives resistance to immunotherapy in CRLM, discusses corresponding strategies for therapeutic remodeling of the immune microenvironment, and examines the clinical trial evidence. **Figure 1** outlines this mechanistic foundation, which spans the underlying CRC immune subtype, the local immunosuppressive hepatic microenvironment, and the systemic hepatic sink effect. In contrast to previous perspectives centered on primary tumors or the overall mCRC population, this review treats CRLM as a liver metastasis-specific disease entity governed by distinctive immunological rules. By integrating mechanisms of resistance, therapeutic countermeasures, and clinical and translational evidence, we aim to provide a framework for liver-microenvironment-directed combination therapy and stratified treatment strategies.

## 2 Mechanistic basis of immunotherapy resistance in CRLM

### 2.1 The intrinsic tolerogenic immune niche of the liver

Organ-specific immune resistance in CRLM is first rooted in the intrinsic immune tolerance of the liver (19). The portal vein continuously delivers gut-derived antigens, microbe-associated molecular patterns, and metabolites to the hepatic sinusoids, keeping the liver under a state of high antigenic load (20). This tolerogenic state is maintained primarily by nonclassical intrahepatic antigen-presenting cells, including Kupffer cells (KCs), liver sinusoidal endothelial cells (LSECs), and hepatic stellate cells (HSCs) (21). KCs are the most abundant innate immune cells in the liver (22). Residing within hepatic sinusoids, they form the first phagocytic barrier for cells and particles arriving through the portal vein (17). Under continuous antigenic and endotoxin stimulation, KCs secrete IL-10 and present antigens with low costimulatory activity, driving contacted CD4+ T cells toward tolerance, suppressing clonal expansion, and supporting local Treg expansion (23). LSECs are fenestrated scavenger endothelial cells lining the sinusoids (24). They constitutively express PD-L1 and can take up circulating antigens and cross-present them to CD8^+^ T cells, but insufficient costimulation directs CD8^+^ T cells toward a hyporesponsive state (25). HSCs reside in the perisinusoidal space and store vitamin A (26). After activation, they secrete TGF-β and retinoic acid, supporting peripheral Treg induction and creating an immunosuppressive fibrotic microenvironment (27). Through low costimulation and the coordinated actions of PD-L1, IL-10, and TGF-β, these cells maintain a tolerogenic state that limits T-cell activation and favors the persistence of FOXP3^+^ Tregs (28). Intrinsic hepatic tolerance therefore precedes tumor colonization, placing CRLM within an unfavorable immune environment from the earliest stages of metastatic formation. This organ-intrinsic permissiveness also outlasts tumor removal. About half of patients recur within two years of curative resection, with the liver involved at first recurrence in approximately two thirds (29, 30). Occult micrometastases persist in macroscopically normal liver in a quiescent stem-like state (31, 32). Quiescence itself confers immune escape, since latency-competent cells impose autocrine WNT inhibition through DKK1 and downregulate the ULBP ligands that trigger NK-cell killing (33). Within the liver, dormancy is enforced by NK cells through IFN-γ, and its release follows contraction of the NK compartment together with accumulation of activated hepatic stellate cells, which secrete CXCL12 and drive NK cells into quiescence through CXCR4 (34). These mechanisms derive from breast and lung models, although the CXCL12-CXCR4 axis they identify is the same one that restrains intrahepatic NK cells within established liver metastases. Hepatectomy with ischemia-reperfusion remodels the remnant liver through neutrophil extracellular trap formation to promote their outgrowth (35). The hepatic niche therefore operates on two timescales, restricting antitumor immunity within established metastases and supporting the survival, immune escape, and later outgrowth of dormant tumor cells after macroscopic disease has been cleared.

### 2.2 Tumor-induced local immunosuppression within liver metastases

The establishment of liver metastases builds on the pre-existing hepatic tolerance with a local program of immune suppression. Within the metastatic lesion, defective dendritic cell priming, dysfunctional effector lymphocytes, and an expanded population of immunosuppressive tumor-associated macrophages limit antitumor immunity and contribute to resistance to immune checkpoint inhibition.

The composition of this local program varies between lesions, so that immune profiles obtained in one clinical context do not necessarily apply to another. Liver metastases grow in two principal histopathological patterns. Desmoplastic lesions, separated from the liver parenchyma by a fibrous rim, carry higher lymphoid infiltration at the invasive margin, whereas replacement lesions, in which tumor cells align along hepatocyte plates and co-opt sinusoidal vessels, show an immune-excluded phenotype with limited T-cell entry into tumor nests and are associated with resistance to antiangiogenic therapy (36–38), although immune density within the tumor core does not differ appreciably between the two patterns (39). Prior treatment is a further source of variation. Intratumoral T-cell density reaches 491 cells/mm² in liver metastases resected within 9.5 weeks of the last oxaliplatin-based cycle, falls to 236 cells/mm² as the interval lengthens, and stands at 292 cells/mm² in chemotherapy-naive lesions, so the chemotherapy-associated increase is transient and reverses (40). Lesions resected after triplet chemotherapy with biologic agents come from a responder-enriched population and are not comparable with an upfront resectable cohort (41). Radiofrequency ablation elicits tumor antigen-specific T-cell responses in peripheral blood without producing appreciable CD8^+^ recruitment into untreated hepatic lesions (42). The prognostically informative immune compartment also differs between synchronous and metachronous disease, although metastatic timing is confounded with treatment exposure and resectability (43, 44). Liver tumor burden and the distinction between *de novo* and recurrent liver metastases await systematic comparison.

#### 2.2.1 Dendritic cell heterogeneity

Dendritic cells occupy the initiating step of the antitumor response, presenting tumor antigens and priming CD8^+^ T cells, and their dysfunction in CRLM limits the effector lymphocyte responses that follow. Dendritic cell dysfunction combines a reduction in conventional dendritic cells that cross-present tumor antigens and prime CD8^+^ T cells with a shift of the remaining dendritic cells toward an immunoregulatory LAMP3^+^ state.

The loss of conventional dendritic cells impairs the priming of tumor-specific CD8^+^ T cells within liver metastases. In orthotopic mouse models of pMMR CRLM, the hepatic lesions contain few dendritic cells and few activated T cells, and this dendritic cell paucity limits the efficacy of immune checkpoint blockade (45). Beyond this numerical reduction, the dendritic cells that remain undergo a functional shift toward a LAMP3^+^ state corresponding to mature regulatory dendritic cells (mregDCs), a conserved state arising from both cDC1 and cDC2 across tumor types (46). These cells retain the maturation and migration markers LAMP3, CCR7, and FSCN1 while co-expressing the inhibitory molecules PD-L1, PD-L2, and IDO1, so that antigen presentation is accompanied by an immunosuppressive program (47, 48). In CRLM single-cell data, LAMP3^+^ dendritic cells express the ligands CD86, PD-L1, and Galectin-9, which are predicted to engage the inhibitory receptors CTLA-4, PD-1, and TIM-3 on dysfunctional, proliferating, and regulatory T cells (49).

The dendritic cell compartment in CRLM therefore combines reduced conventional dendritic cell priming with an immunoregulatory LAMP3^+^ program, linking defective priming, regulatory T cell interactions, and local checkpoint-mediated suppression.

#### 2.2.2 Effector lymphocyte dysfunction

Within established liver metastases, effector lymphocyte responses are restricted at multiple stages downstream of antigen priming. This dysfunction is centered on CD8^+^ T cells and also involves innate-like effector lymphocytes, including NK cells, γδ T cells, and ILC1-like cells. Effector lymphocytes are restricted at each stage from infiltration through local suppression to tissue maintenance, and this restriction is further modulated by the gut-liver metabolic axis.

Effector T cells are underrepresented within liver metastases. Paired MSS samples show that the proportions of total CD3^+^ T cells and CD3^+^CD8^+^ cytotoxic T cells are lower in the central region of liver metastases than in paired primary tumors, while CD33^+^ myeloid suppressor cells are enriched, indicating that effector T cells are depleted specifically within the tumor core (50).

The infiltrating effector T cells are further restrained by enhanced PD-L1 signaling. PD-L1 expression is higher in liver metastases than in paired primary tumors in patients with synchronous metastases or concurrent resection, indicating that insufficient effector T-cell infiltration is accompanied by inhibitory ligand upregulation (51). This increase can be traced to tumor cell-intrinsic regulation. Matrix Gla protein (MGP) and chondroitin sulfate synthase 1 (CHSY1) are both upregulated in liver metastases relative to paired primary tumors and converge on the PD-L1 promoter through NF-κB and HIF1α signaling respectively, driving CD8^+^ T-cell exhaustion-like phenotypes marked by PD-1, LAG-3, and TIM-3 (52). Loss of CHSY1 restores the proportion of CD8^+^CD103^+^ tissue-resident memory T cells (53).

The durability of effector immunity is further limited by insufficient maintenance of tissue-resident CD8^+^ T cells. CD8^+^CD103^+^ tissue-resident memory T cells participate in local immune surveillance and rapid cytotoxic responses, and a reduction in this population indicates that liver metastases may lack T-cell subsets capable of long-term retention and sustained local killing (54–56). A higher abundance of CD103^+^ tumor-infiltrating lymphocytes within liver metastases associates with better patient prognosis, and CD103^+^ tumor-infiltrating lymphocytes in primary tumors show no such association, indicating that inadequate tissue-resident T-cell immunity within liver metastases links to poorer outcomes in CRLM (57).

NK cells are abundant in the liver and represent a potential effector arm against CRLM, yet their antitumor activity is limited both by poor recruitment into the metastatic tissue and by functional restraint of the NK cells that remain. At the level of recruitment, NK cells enter metastatic tissue inefficiently and adopt a tissue-imposed phenotype distinct from circulating NK cells, indicating that the hepatic niche governs their state (58, 59). The recruitment and residency of CD49a^+^CD49b^+^ ILC1-like NK cells in CRLM depend on CXCL9, CXCL10, and CXCR3 signaling, and NKp46^+^ cell-specific deletion of Cxcr3 increases liver metastatic burden (60). The NK cells that do reside in the liver are heterogeneous and retain antitumor capacity. Single-cell analysis of CRLM resolves an abundant intrahepatic CD56bright population carrying a CXCR6^+^EOMES^+^CD69^+^ liver-residency program, distinct from a CD56dim subset that retains circulating and cytotoxic features (61), and NK phenotype is governed more by tissue of origin than by tumor status when compared across primary tumor, adjacent non-tumoral liver, liver metastasis, and peripheral blood (62). Both subsets are held in check by targetable inhibitory pathways. CXCL12 acting through CXCR4 lowers IFN-γ output by intrahepatic CD56dim NK cells, and the CXCR4 antagonist plerixafor restores this response (61). IL-1R8 acts as an innate checkpoint whose genetic deletion enhances NK maturation and IL-18-dependent control of liver metastasis in mice, and whose antibody blockade increases TNF-α and IFN-γ production by intrahepatic CD56dim NK cells from patients with CRLM (61, 63). CXCR4 and IL-1R8 therefore represent candidate targets for restoring NK-mediated surveillance in ICI-resistant liver metastases.

γδ T cells form a context-dependent unconventional T-cell compartment in CRLM. Intrahepatic CD69^+^ Vδ1 T cells show tissue-resident effector features, recirculate into peripheral blood with liver-related transcriptional and TCR signatures, and are associated with slower metastatic progression and longer overall survival (64). Liver metastases also dampen IL-18-driven Vδ1 γδ T cell activity through the IL-18/IL-18BP axis, linking organ-specific cytokine regulation to reduced immunotherapy responsiveness (65). γδ T cells therefore contribute to both tissue-resident cytotoxic surveillance and cytokine-restricted dysfunction in CRLM.

The gut-liver metabolic axis shapes effector lymphocyte function in liver metastases through bile acid metabolism. Chronic stress reduces intestinal bacteria expressing bile salt hydrolase (BSH) and alters bile acid composition, raising PD-1 and LAG-3 expression on intrahepatic CD8^+^ T cells and impairing their effector function. Supplementation with hyocholic acid partially restores the proportion of IFN-γ^+^ CD8^+^ T cells through TGR5 (66). Bile acid signaling therefore acts as an upstream control point for CD8^+^ T-cell hyporesponsiveness within liver metastases.

Effector lymphocyte dysfunction in CRLM is therefore a continuous defect spanning restricted infiltration, inadequate tissue residency, and constrained innate-like effector function, which together prevent durable cytotoxic responses within liver metastases.

#### 2.2.3 Heterogeneity and suppressive programs of tumor-associated macrophages

Tumor-associated macrophages (TAMs) are the dominant immune population within liver metastases. Their phenotypes have traditionally been described along the M1 and M2 axis, in which M1-like cells sustain inflammatory and antitumor responses and M2-like cells sustain tissue repair and immune suppression. Human CRLM studies additionally resolve TAMs by morphology, molecular state, and spatial position. In CRLM, TAMs separate into small (S-TAM) and large (L-TAM) populations identifiable on routine CD163 immunohistochemistry, and mean CD163^+^ TAM area is associated with recurrence, whereas the association with CD163^+^ cell density is weaker in the same cohort (67). L-TAMs express Kupffer-cell genes (CD5L, MARCO, VCAM1) together with a lipid-rich, LXR-driven program and complement components (C1Q), and S-TAMs express an inflammatory monocyte-associated signature (S100A8/9, FCN1). These origins are inferred from transcriptional profiles and await confirmation by lineage tracing (67, 68).

Beyond this morphological distinction, single-cell studies resolve CRC and CRLM macrophages into discrete molecular states (69, 70). A pro-tumor SPP1^+^ state and a complement-high C1QC^+^ state represent the principal axis, and both derive from tumor-infiltrating FCN1^+^ monocyte-like precursors (69). The SPP1^+^ state is enriched in angiogenesis and in metastatic liver cancer pathways, and the C1QC^+^ state is enriched in antigen-presenting and complement programs. A low C1QC^+^ together with a high SPP1^+^ signature predicts the worst prognosis. A proliferating MKI67^+^ PCNA^+^ signature marks a subset that is preferentially depleted by anti-CSF1R blockade (69). These states are distributed unevenly within the lesion, and their position tracks with outcome. A mature TAM population transcriptionally close to Kupffer cells and marked by GPNMB accumulates at the tumor edge and extends into the tumor center, where it is more exposed to immunosuppressive signals, suppresses CD8^+^ T cell responses through IL-10, expresses CD274, and predicts shorter disease-free and overall survival, and GPNMB itself inhibits T cell activation and correlates with reduced response to ICIs. Inflammatory monocyte-derived macrophages marked by SERPINB2 distribute evenly across the invasive margin and associate with longer disease-free survival (70).

These macrophage states carry a network of inhibitory receptors and secreted programs. Paired single-cell profiling of primary CRC and matched liver metastases shows that the liver lesions are enriched for immunoregulatory TAM states expressing suppressive molecules (71). CD11b^+^ CTLA-4^+^ myeloid cells drive tumor evasion in CRC through a CTLA-4 to IL-1β axis, and combined blockade of CTLA-4 and IL-1β suppresses metastasis in anti-PD-1-resistant models (72). TIM-3 and VISTA are upregulated on macrophages in a mesenchymal subgroup of MSS CRLM and contribute to their immunosuppressive phenotype (73, 74). FSTL1 induces CD11b^+^ DIP2A^+^ LAG-3^+^ myeloid cells that promote T-cell dysfunction in CRC (75). VSIG4 carries particular relevance to the liver, since this co-inhibitory molecule, predominantly expressed by Kupffer cells, calibrates CD8^+^ T-cell receptor signaling through an interaction with CD5 that favors the outgrowth of poorly immunogenic metastatic clones, and nanoantibody blockade of VSIG4 sensitizes liver metastases to anti-PD-L1 therapy (76). IDO is expressed in CRLM together with PD-1, PD-L1, and CD163^+^ TAMs, and IDO1 and COX2 coexpression predicts poor survival in CRC liver oligometastases (77, 78). TGF-β limits peripheral memory CD8^+^ T-cell recruitment and instructs SPP1^+^ TAMs to restrict the clonal expansion of newly recruited T cells in liver metastases (79). Macrophage-centered suppression in CRLM therefore involves multiple checkpoint, metabolic, and cytokine programs, of which PD-L1 is one component.

### 2.3 The hepatic sink effect

Beyond impairing local antitumor immunity, CRLM reshapes systemic immune responses and reduces the sensitivity of extrahepatic tumors to ICIs, a phenomenon termed the hepatic sink effect. Clinical studies first indicated that liver metastases and extrahepatic metastases respond differently to immunotherapy-based combinations. In the REGOTORI study, among patients with MSS/pMMR mCRC treated with regorafenib plus toripalimab, the ORR was 100% in patients with lung-only metastases but 0% in those with liver-only metastases, and no objective responses were observed in patients with both lung and liver metastases (80). One route for this organ-specific effect lies in the hepatic vasculature, since paired scRNA-seq and spatial validation show that ACKR1+ endothelial cells colocalize with CCL5+CD8+ T cells around hepatic sinusoidal vessels in CRLM, a configuration that may support the retention of effector T cells within the liver (49).

Liver metastases can reduce systemic effector T-cell numbers through myeloid cell-mediated T-cell elimination (13). In clinical cohorts and mouse models, liver metastases reduced ICI efficacy and lowered T-cell numbers in blood and tumors, and in mice they impaired anti-PD-L1 responses at both subcutaneous and hepatic sites, whereas lung metastases did not. Mechanistically, activated CD8^+^ T cells are retained in the liver through LFA-1/CD44-mediated adhesion and are then deleted by CD11b^+^F4/80^+^ monocyte-derived macrophages through FasL-Fas signaling. Depletion of intrahepatic myeloid cells or liver-directed radiotherapy reduces intrahepatic T-cell apoptosis, restores effector T-cell infiltration in extrahepatic lesions, and reinstates the efficacy of anti-PD-L1 therapy against extrahepatic tumors (13).

In addition to eliminating effector T cells through myeloid cells, liver metastases can suppress T-cell responses in distant lesions through a Treg-MDSC axis. Single-cell atlases of CRLM show a reduced proportion of CD8^+^ T cells and enrichment of Tregs and myeloid suppressor cells within liver metastases, indicating the formation of a local suppressive network centered on myeloid cells and Tregs (81). Liver lesions markedly reduce the responsiveness of distant subcutaneous tumors to anti-PD-1 therapy, whereas lung, kidney, or intraperitoneal lesions of comparable burden do not. Injection of irradiated MC38 cells or MC38 tumor lysate into the liver also promotes the progression of distant MC38 subcutaneous tumors, whereas injection of unrelated B16F10 antigens into the liver does not produce the same effect, indicating that this process is both organ-specific and antigen-specific (16). Mechanistically, liver metastasis induces highly activated Tregs that reduce CD80 and CD86 on CD11b^+^ monocytes through CTLA-4-mediated trans-endocytosis, promote MDSC expansion in distant tumor microenvironments, and deprive effector T cells of adequate costimulatory signals. Selective depletion of Tregs or treatment with Treg-depleting anti-CTLA-4 combined with anti-PD-1 restores effector T-cell responses in distant tumors (16). The hepatic sink effect therefore combines effector T-cell retention and deletion with distant costimulatory suppression, so that CRLM also reduces the treatment sensitivity of extrahepatic lesions.

## 3 Therapeutic remodeling strategies for the CRLM immune microenvironment

Resistance in CRLM arises from these mechanistic layers acting together, so therapeutic remodeling is best directed at them collectively, by increasing tumor immunogenicity, restoring effector lymphocyte function through relief of myeloid and Treg suppression, improving stromal and vascular accessibility, and counteracting the hepatic sink effect. These strategies differ markedly in translational maturity, since randomized evidence obtained specifically in CRLM populations is currently lacking and available support ranges from single-arm clinical activity to preclinical data. Their rationale, supporting evidence, and key findings in CRLM are summarized in **Table 1**, and their correspondence with the mechanistic layers described in Section 2 is outlined in **Figure 1**.

**Table 1:** Therapeutic and translational strategies for remodeling the CRLM immune microenvironment.

Strategy	Immunological rationale	Evidence/model	Key findings in CRLM	References
Oxaliplatin-based immunogenic cell death with nano-delivery	Induces immunogenic cell death; increases antigen release and DC activation; increases CD8^+^ T-cell infiltration; reduces MDSCs, M2-like macrophages and Tregs	Preclinical CRC liver-metastasis model; Nano-Folox/5-FU with anti-PD-L1	Converted pMMR liver metastases from immune-cold to inflamed; restored anti-PD-L1 activity	Guo et al., 2020 (82)
Chemotherapy timing and immune-state remodeling	Chemotherapy exposes antigen and reshapes immunity, but mistimed hepatic exposure worsens T-cell attrition and myeloid suppression	Translational/preclinical	CRLM sensitization depends on treatment sequence and timing, not drug choice alone	Ma et al., 2023 (83)
Local ablation as *in situ* immune sensitization	Tumor necrosis and DAMP release recruit CD8^+^ cytotoxic T cells and induce distant (abscopal) effects	Preclinical CT26 flank model (unresectable CRLM); ethanol ablation and radiofrequency ablation	Increased local and contralateral CD8^+^ cytotoxic T-cell density; not yet validated orthotopically	Yang et al., 2026 (84)
Photodynamic therapy for MSS CRLM	ROS-mediated tumor injury reduces macrophage-associated suppression and enhances T-cell costimulation	Preclinical/local therapy	Local immune-sensitizing rationale where systemic ICI alone is weak	Xu et al., 2025 (85)
TEC triplet regimen	Combines cytotoxic therapy, immune checkpoint blockade and ADCC to link antigen release with effector recovery	Clinical/translational; MSS mCRC with liver metastases	Preliminary activity in a difficult MSS liver-metastatic population	Xu et al., 2024 (89)
Chemokine-axis blockade against suppressive myeloid recruitment	Targets NLRP6–CCL2–CCR2, CCL4–CCR5 and TWEAK–Fn14 signaling to reduce MDSC, TAM and Treg recruitment and tumor invasion	Preclinical mechanistic	Provides targetable axes linking tumor-intrinsic or inflammatory signaling to myeloid-dominant suppression	Chang et al., 2024 (90); Liu et al., 2024 (91)
TAM reprogramming (IFN-α plus anti-CTLA-4)	Reprograms macrophages toward antigen presentation and expands tumor-reactive CD8^+^ T-cell clones; CTLA-4 blockade further amplifies priming	Mechanistic preclinical combination	Myeloid remodeling can convert CRLM from suppressive to responsive	Kerzel et al., 2023 (92)
Innate immune restoration via STING–NLRP3–IL-18	Maintains macrophage–NK 4-1BBL/4-1BB signaling and supports intrahepatic NK-cell cytotoxicity	Preclinical CRLM innate-immunity study	Option for low-T-cell CRLM where T-cell-centered ICI is insufficient	Sun et al., 2023 (95)
Stromal softening and vascular-access improvement	Reduces CAF activity, collagen deposition and ECM crosslinking to improve CD8^+^ T-cell entry and drug/antibody access	Nanomedicine with anti-PD-L1; stromal/vascular reprogramming	Sonodynamic nanotherapy increased CD8^+^ T-cell entry into tumor cores; RAS inhibition improved bevacizumab efficacy	Huang et al., 2023 (99); Shen et al., 2020 (98)
Treg-depleting anti-CTLA-4 against the hepatic sink	Relieves Treg-mediated costimulatory suppression and reverses MDSC expansion in distant lesions	Dual-site mouse models; organ- and antigen-specific	Treg-depleting anti-CTLA-4 plus anti-PD-1 restored effector responses in distant tumors	Lee et al., 2020 (16)
IL-10R blockade against systemic liver-driven suppression	Interrupts the Treg–IL-10–monocyte–PD-L1 axis and reduces systemic suppressive signaling	Mechanistic/therapeutic intervention	Supports targeting suppressive cytokine circuitry rather than checkpoint receptors alone	Shiri et al., 2024 (100)
SPHK1 inhibition plus anti-PD-1 and liver-directed radiotherapy	Targets SPHK1^+^ TAM-driven S1P/NLRP3/IL-1β signaling and reduces exhaustion-associated CD8^+^ T-cell states	Preclinical/translational CRLM	Links myeloid inflammasome signaling, PD-1 resistance and liver-directed radiation into one strategy	Zhan et al., 2025 (101)
Liver-directed radiotherapy plus dual immune checkpoint blockade	Radiation releases antigen and remodels liver lesions while durvalumab/tremelimumab amplify T-cell responses	Clinical study	Clinical exploration of abscopal activation; responses were limited	Segal et al., 2021 (102)
Hepatocyte-directed organ-level remodeling	ASGPR-mediated miR-122 delivery downregulates prometastatic and immunosuppressive programs in nontumor liver	Preclinical hepatocyte-targeted nanomedicine	Increased intrahepatic CD8^+^ T cells and reduced MDSCs; organ-level remodeling beyond tumor-cell targeting	Sendi et al., 2022 (103)

ADCC, antibody-dependent cellular cytotoxicity; ASGPR, asialoglycoprotein receptor; CAF, cancer-associated fibroblast; CRC, colorectal cancer; CRLM, colorectal cancer liver metastasis; CTLA-4, cytotoxic T-lymphocyte-associated protein 4; DAMP, damage-associated molecular pattern; DC, dendritic cell; ECM, extracellular matrix; ICD, immunogenic cell death; ICI, immune checkpoint inhibitor; IFN, interferon; IL, interleukin; mCRC, metastatic colorectal cancer; MDSC, myeloid-derived suppressor cell; MSS, microsatellite stable; NK, natural killer; PD-1, programmed cell death protein 1; PD-L1, programmed death-ligand 1; pMMR, mismatch repair-proficient; RAS, rat sarcoma; ROS, reactive oxygen species; S1P, sphingosine-1-phosphate; SPHK1, sphingosine kinase 1; STING, stimulator of interferon genes; TAM, tumor-associated macrophage; Treg, regulatory T cell.

Increasing tumor immunogenicity is the initiating step of immune remodeling in CRLM. Oxaliplatin-containing regimens can increase tumor antigen exposure by inducing immunogenic cell death, and nano-delivery approaches such as Nano-Folox further improve drug accumulation and immune activation within tumor tissues (82). Chemotherapy-induced remodeling is temporally dependent. In a preclinical model, oxaliplatin given before metastatic colonization reduced intrahepatic T cells and reshaped the liver into a pro-metastatic niche (83). The timing of chemotherapy relative to colonization is therefore a key determinant of immune sensitization. Beyond systemic therapy, intratumoral chemical ablation can increase CD8^+^ T-cell infiltration through local necrosis and antigen release and induce abscopal immune responses in contralateral lesions (84). Photodynamic therapy can reduce macrophage-associated suppressive signals and enhance T-cell costimulatory molecule expression through ROS-mediated tumor injury, providing a rationale for local immune sensitization in MSS CRLM (85).

Increasing immunogenicity alone is usually insufficient to overcome the multilayered suppression of CRLM. Immune sensitization therefore needs to be connected with strategies that amplify effector immunity. Although epigenetic modulation, MEK inhibition, or oncolytic viruses combined with ICIs can theoretically enhance antigen presentation, interferon responses, or tumor inflammation, current clinical and translational evidence shows limited efficacy, suggesting that single-point immune sensitization is unlikely to fully reverse the tolerogenic microenvironment of liver metastases (86–88). The TEC triplet regimen, comprising tislelizumab, cetuximab, and irinotecan, can serve as a representative combination linking enhanced immunogenicity with restoration of effector immunity (89). Within this regimen, irinotecan, a topoisomerase I inhibitor, provides cytotoxic chemotherapy that induces tumor-cell DNA damage and immunogenic cell death, thereby promoting tumor antigen release; tislelizumab, an anti-PD-1 antibody, delivers immune checkpoint blockade that relieves inhibitory signaling and restores effector T-cell activation; and cetuximab, an anti-EGFR IgG1 monoclonal antibody, blocks EGFR-driven proliferative signaling while engaging NK cells and macrophages through its Fc region to mediate antibody-dependent cellular cytotoxicity (ADCC). This combination has shown preliminary efficacy in MSS disease with liver metastases (89). It acts mainly on antigen release and effector activation, leaving myeloid and Treg suppression, stromal and vascular barriers, and the systemic effects of the hepatic niche to be addressed.

Cellular suppression in CRLM can be relieved at several levels, including the recruitment of suppressive cells, their functional state, innate immune support, and tumor-derived regulation. At the level of recruitment, blocking chemokine-receptor signaling reduces the trafficking of MDSCs, TAMs and Tregs into CRLM lesions; interference with the NLRP6-CCL2-CCR2 axis reduces M-MDSCs and increases CD8+ T-cell infiltration, and CCL4-CCR5 and TWEAK-Fn14 signaling provide additional candidate targets for limiting Th17-cell recruitment and tumor invasion (90, 91). At the level of functional reprogramming, engineered TAMs expressing IFN-α enhance antigen presentation and expand tumor-reactive CD8+ T-cell clones, and their combination with anti-CTLA-4 increases the complete response rate, indicating that reprogramming resident TAMs can convert myeloid cells from a suppressive to a stimulatory state (92). Stachydrine-mediated JAK2-STAT3 inhibition and ginsenoside Rb1-mediated attenuation of the circ_0034880-SPP1 axis act similarly but rest on preclinical data (93, 94). At the innate immune level, the STING-NLRP3-IL-18 axis sustains 4-1BBL/4-1BB signaling between macrophages and NK cells and supports intrahepatic NK cytotoxicity, offering a T cell-independent strategy for CRLM with low T-cell infiltration (95). At the level of tumor-derived regulation, ENO2-MIF and Wnt-BCL9 signaling and tumor epithelial suppression of dendritic cell function are candidate targets for restoring effector immunity (81, 96, 97). Most of this evidence derives from mouse models and single-cell analyses, and its translational value in human CRLM remains to be confirmed in patient-derived samples and liver-lesion biopsies.

Improving stromal and vascular barriers may increase the sensitivity of CRLM to ICIs by facilitating effector T-cell entry and function within liver metastatic lesions. Cancer-associated fibroblasts, matrix deposition, collagen crosslinking, and abnormal vasculature restrict CD8+ T-cell migration toward the tumor center, so stromal softening and vascular normalization are strategies for restoring immune accessibility. RAS inhibition reduces metastasis-associated fibroblast activity and collagen crosslinking and improves the efficacy of bevacizumab (98). In addition, inhibition of TGF-β receptor I can reduce CAF abundance and collagen deposition, while sonodynamic therapy can induce immunogenic cell death and promote local immune activation. When combined with anti-PD-L1 therapy, this combined remodeling strategy promotes CD8^+^ T-cell entry into the metastatic tumor center and enhances antitumor immune activation (99).

Where the influence of liver metastases reaches extrahepatic sites, treatment also needs to counteract the suppressive effect of the hepatic sink on systemic antitumor immunity. Treg-depleting anti-CTLA-4 combined with anti-PD-1 restores effector T-cell responses in distant tumors in dual-site liver metastasis models, indicating that relieving Treg-MDSC-mediated costimulatory suppression is a key strategy for counteracting the hepatic sink effect (16). IL-10R blockade can interfere with the Treg-IL-10-monocyte-PD-L1 axis, reduce systemic suppressive signals, and enhance immunotherapy efficacy (100). SPHK1 inhibition combined with anti-PD-1 and liver-directed radiotherapy can reduce intrahepatic immune suppression and improve control of distant lesions (101). Radiotherapy (most commonly liver-directed, 29% of patients) combined with durvalumab and tremelimumab can induce limited abscopal responses and provides a clinical exploratory basis for radiotherapy plus dual immune blockade to counteract the hepatic sink effect (102). In addition, asialoglycoprotein receptor (ASGPR)-mediated hepatocyte-targeted delivery of miR-122 can downregulate pro-metastatic and immunosuppression-associated factors in nontumor liver tissue, increase the intrahepatic CD8^+^ T-cell proportion, and reduce the MDSC proportion (103), indicating that hepatocyte-directed remodeling may also serve as an organ-level strategy to counter liver-derived immune suppression.

Because no immune-remodeling strategy has yet been validated in a randomized CRLM-specific trial, and most remain preclinical or early translational, the priority is to advance the most mature combinations into liver-metastasis-specific testing. In such studies, liver-lesion response, paired biopsies, and post-treatment immune profiling should become the criteria for determining whether treatment truly remodels the hepatic microenvironment.

## 4 Clinical trials of immunotherapy in CRLM

In CRLM, the efficacy of ICIs is shaped by the molecular subtype of the underlying tumor and by the liver metastases themselves, and liver involvement emerges as a consistent adverse factor across trials. Trial designs, regimens, and liver-metastasis-related outcomes are summarized in **Table 2**.

**Table 2:** Clinical trials of immune checkpoint inhibitor–based therapy in colorectal cancer liver metastasis.

Trial/registration	Design/phase	Subtype	Regimen	N	Overall efficacy	Liver-metastasis–related outcome	Reference
NCT03946917	Phase Ib/II, single-arm	MSS/pMMR	Regorafenib + toripalimab	42	ORR low overall; mPFS 2.1 mo; mOS 15.5 mo	Liver-only ORR 0% (0/4) vs lung-only 100% (3/3); with-liver ORR 8.7% vs no-liver 30%; liver-only mPFS 2.5 vs lung-only 11.4 mo	Wang et al., 2021 (80)
NCT03860272	Phase 1, escalation + expansion	MSS/pMMR	Botensilimab + balstilimab	148 (101 evaluable)	No active liver mets: ORR 22%, DCR 73%, mPFS 4.1 mo, mOS 20.9 mo	Active liver mets: ORR 0% (95% CI 0–14), mPFS 1.4 mo, mOS 7.4 mo; all confirmed responders had no active liver mets	Bullock et al.,2024 (15)
NCT02873195	Phase II, randomized, double-blind, placebo-controlled	MSS/pMMR 89%	Capecitabine + bevacizumab ± atezolizumab	128 (mITT)	mPFS 4.4 vs 3.6 mo, HR 0.75 (95% CI 0.52–1.09)	With-liver ORR 5.8% vs no-liver 23.1% (P = 0.04); OS benefit confined to no-liver (interaction P = 0.04; no-liver HR 0.33 vs liver HR 1.14)	Mettu et al.,2022 (105)
NCT02060188	Phase II, open-label, non-randomized	MSI-H/dMMR	Nivolumab + relatlimab (LAG-3)	50	Investigator-assessed ORR (RECIST v1.1), primary endpoint	With-liver ORR numerically lower than no-liver; per-subgroup figures reported in supplement only	Overman et al.,2024 (104)
NCT03202758	Phase Ib/2, single-arm, multicenter	RAS-mutant; MSS (n=48)	mFOLFOX6 + durvalumab + tremelimumab	57 (MSS 48)	Primary 3-month PFS endpoint (MSS); median follow-up 36 mo	Liver mets 79%; presence of liver mets not a significant factor for PFS or ORR (forest plot, NS)	Thibaudin et al.,2023 (106)
NCT03721653	Phase II, randomized, open-label, first line	pMMR 91%	FOLFOXIRI + bevacizumab ± atezolizumab	218 (ITT)	mPFS 13.1 vs 11.5 mo, HR 0.69 (80% CI 0.56–0.85); ITT ORR 59% vs 64%	Liver-limited disease: PFS interaction P = 0.87, subgroup HR 0.72 (95% CI 0.38–1.37)	Antoniotti et al.,2022 (107)
ChiCTR2000035642	Phase II, single-arm, single-center	MSS, RAS/BRAF WT	Tislelizumab + cetuximab + irinotecan	33 (mITT)	ORR 33%, DCR 79%, mPFS 7.3 mo, mOS 17.4 mo	With-liver ORR 38.5% (10/26) vs no-liver 33.3% (P>0.99); with-liver mPFS 6.1 vs no-liver 8.6 mo (P = 0.38)	Xu et al.,2024 (89)
ChiCTR2100046768	Phase II, open-label, single-arm	MSS/pMMR	FMT + tislelizumab + fruquintinib	20	ORR 20%, mPFS 9.6 mo	With-liver mPFS 5.6 mo, mOS 10.6 mo vs no-liver not reached; liver mets independent adverse PFS factor	Zhao et al.,2023 (110)
NCT05291156	Phase II, randomized, open-label	MSS, RAS/BRAF WT	Cetuximab ± avelumab (rechallenge)	156	Primary endpoint OS	No-liver subgroup favored avelumab arm (PR 11% vs 0%; mPFS 6.0 vs 4.3 mo); with-liver subgroup no difference	Ciardiello et al.,2026 (111)
NCT02851004	Phase I/II, single-arm, two cohorts	MSI-H (A) and MSS (B)	Napabucasin + pembrolizumab	55	Cohort A (MSI-H) irORR 50%; cohort B (MSS) irORR 10%, mPFS 1.6 mo, mOS 7.3 mo	Liver mets 70% of MSS cohort; no liver-specific ORR reported	Kawazoe et al.,2020 (108)
NCT02260440	Phase II, single-arm; terminated for futility	Mostly pMMR	Pembrolizumab + azacitidine	30 treated	ORR 3%, mPFS 1.9 mo, mOS 6.2 mo	No liver-specific analysis reported	Kuang et al.,2022 (86)
NCT03206073	Phase I/II, four cohorts	MSS/pMMR	Pexastimogene devacirepvec + durvalumab ± tremelimumab	34 (25 evaluable)	durvalumab arm DCR 12.5%, mPFS 2.3 mo; treme/durva arm DCR 16.7%, mPFS 2.1 mo	No liver-specific ORR/PFS reported	Monge et al.,2023 (88)
NCT03374254	Phase Ib, non-randomized, open-label	MSS/pMMR	Pembrolizumab + binimetinib ± mFOLFOX7 or FOLFIRI	51	ORR cohort A 0%, C 9%, E 15%; mPFS 2.2/12.2/6.1 mo	No liver-specific subgroup analysis reported	Chen et al.,2024 (87)
NCT03122509	Phase II, single-arm	MSS/pMMR	Durvalumab + tremelimumab + concurrent radiotherapy	24	ORR 8.3% (non-irradiated lesions), mPFS 1.8 mo, mOS 11.4 mo	Liver was the most common irradiated site (29%); no liver-specific ORR reported	Segal et al.,2021 (102)
NCT03274804	Phase I, single-arm	MSS/pMMR	Pembrolizumab + maraviroc (CCR5)	20	ORR 5.3%, mPFS 2.1 mo, mOS 9.8 mo	Liver mets 75%; paired liver biopsies feasible in 94.7%, post-treatment T-cell aggregation with IFN-γ/CXCL9/CXCL10 upregulation despite ORR 5.3%	Haag et al.,2022 (109)

Trials are ordered by the strength of liver-metastasis–specific data. Statistics (P values, hazard ratios, confidence intervals) are retained in the table and omitted from the main text. CI, confidence interval; DCR, disease control rate; HR, hazard ratio; ICI, immune checkpoint inhibitor; ITT, intention-to-treat; mITT, modified intention-to-treat; mOS, median overall survival; mPFS, median progression-free survival; MSS, microsatellite stable; NS, not significant; ORR, objective response rate; pMMR, mismatch repair-proficient; WT, wild type.

In MSI-H/dMMR disease, liver metastases reduce the benefit of ICIs. In a first-line pembrolizumab cohort, median progression-free survival was 6 versus 34 months and the objective response rate 21% versus 63% in patients with and without liver metastases (14). In the CheckMate 142 cohort treated with nivolumab plus relatlimab, the response rate in patients with liver metastases was numerically lower than in patients without liver involvement (104).

In MSS/pMMR disease, the adverse effect of liver metastases is more pronounced. In a phase 1 trial of botensilimab plus balstilimab, the objective response rate was 0% versus 22% and median overall survival 7.4 versus 20.9 months in patients with and without active liver metastases (15). In the REGOTORI study of regorafenib plus toripalimab, the response rate reached 100% in patients with lung-only metastases and 0% in patients with liver-only metastases (80). In the BACCI trial of capecitabine and bevacizumab with or without atezolizumab, the response rate was 5.8% versus 23.1%, and the overall survival benefit was confined to patients without liver involvement (105).

Several chemotherapy-based combinations retained activity in the presence of liver metastases. In the MEDITREME trial of mFOLFOX6 with durvalumab and tremelimumab, progression-free survival and response were similar in patients with and without liver metastases (106). In the AtezoTRIBE trial of FOLFOXIRI and bevacizumab with or without atezolizumab, the progression-free survival benefit was similar in patients with liver-limited disease (107). In a phase 2 trial of tislelizumab, cetuximab, and irinotecan, the response rate was comparable in patients with liver metastasis (38.5%) versus those without liver metastasis (33.3%) (89). These results indicate that an active chemotherapy backbone can preserve efficacy when liver metastases are present.

Combinations designed to increase immunogenicity or to relieve a single suppressive signal showed limited activity in CRLM. Napabucasin plus pembrolizumab produced a response rate of 10% (108). Pembrolizumab plus azacitidine produced a response rate of 3% and was terminated for futility (86). Pexastimogene devacirepvec combined with durvalumab and tremelimumab produced a disease control rate below 20% (88). Pembrolizumab plus binimetinib with or without chemotherapy produced response rates between 0% and 15% (87). Durvalumab and tremelimumab with concurrent radiotherapy produced a response rate of 8.3% (102). These results indicate that single-point immune sensitization is insufficient in MSS CRLM. Two contributions operate in this setting, the low immunogenicity of MSS/pMMR disease, which applies at any metastatic site, and the additional restriction imposed by the liver metastatic niche. Evidence obtained outside these trials establishes the second as a distinct contribution. Liver involvement also shortens progression-free survival and lowers response in MSI-H/dMMR disease, where tumor immunogenicity is high and immune checkpoint inhibitors are otherwise effective (14), and a hepatic lesion abolishes the response of a distant subcutaneous tumor derived from the same cell line while a lung lesion of comparable burden leaves it intact (16). These trials report response at the patient level, so the relative weight of the two contributions remains to be measured, and liver-lesion response together with paired intrahepatic sampling would supply that measure.

A shared limitation is the scarcity of liver-lesion-specific outcomes, since most trials report only the presence or absence of liver metastases, and seldom report liver-lesion response, progression-free survival, or conversion-to-resection rates. In the PICCASSO trial, the treatment feasibility rate (its primary endpoint) was 94.7%, while serial liver-metastasis biopsies obtained in a subset of patients showed intratumoral T-cell aggregation with upregulation of IFN-γ, CXCL9, and CXCL10, while the objective response rate was 5.3% (109). Intrahepatic immune activation therefore does not consistently correspond to radiographic response. Current evidence remains insufficient to determine whether immunotherapy overcomes liver-specific resistance.

## 5 Discussion and conclusion

This review has approached CRLM as a liver metastasis-specific disease entity. Organ-specific resistance arises from three connected layers, comprising an intrinsic tolerogenic niche that precedes tumor colonization, a local microenvironment that after colonization suppresses antitumor immunity through myeloid, dendritic cell, and lymphocyte programs, and a hepatic sink effect that extends this suppression systemically. These layers explain why liver involvement is consistently associated with resistance across both MSI-H/dMMR and MSS/pMMR contexts, why multitarget remodeling directed at the hepatic niche is more promising than single sensitizing interventions, and why an active chemotherapy backbone preserves efficacy where single-point immune sensitization does not.

However, current evidence remains insufficient to determine whether this organ-specific resistance can be reversed. Most mechanistic and therapeutic findings derive from preclinical models and from cohorts representing the overall mCRC population, and direct validation at the level of liver metastatic lesions is still lacking. Reported trials seldom provide liver-lesion-specific outcomes, and intrahepatic immune activation does not consistently correspond to radiographic response, so it remains unclear whether liver-specific resistance can be overcome in patients.

Future research should therefore be designed specifically around liver metastases. Studies should prespecify a three-tier classification of liver metastatic burden, adopt liver-lesion ORR, liver-lesion PFS, conversion-to-resection rate, and paired biopsies as major endpoints, and combine single-cell analysis, spatial transcriptomics, and liquid biopsy into an integrated predictive framework spanning imaging, tissue, and peripheral blood. Pursuing these directions would establish CRLM as an organ-specific disease entity with distinctive immunological rules and advance CRC immunotherapy from a primary-tumor-centered approach toward a liver-microenvironment-oriented approach.
